# Supplementary material for: Focused ultrasound ablation of melanoma with boiling histotripsy yields abscopal tumor control and antigen-dependent dendritic cell activation
Source: Theranostics. 2024 Feb 11;14(4):1647–61. doi: 10.7150/thno.92089 (PMC10879863; doi:10.7150/thno.92089)
Supplement: Supplementary file 1 — Supplementary figures and tables. [file thnov14p1647s1.pdf]

Supplementary Materials for

Focused ultrasound ablation of melanoma with boiling histotripsy yields abscopal tumor control and antigen-dependent dendritic cell activation

Eric A Thim *et al.*

*Corresponding author: Richard J. Price, rprice@virginia.edu & Timothy N. J. Bullock, tb5v@virginia.edu

This PDF file includes:

Figs. S1 to S8
Tables S1 to S2

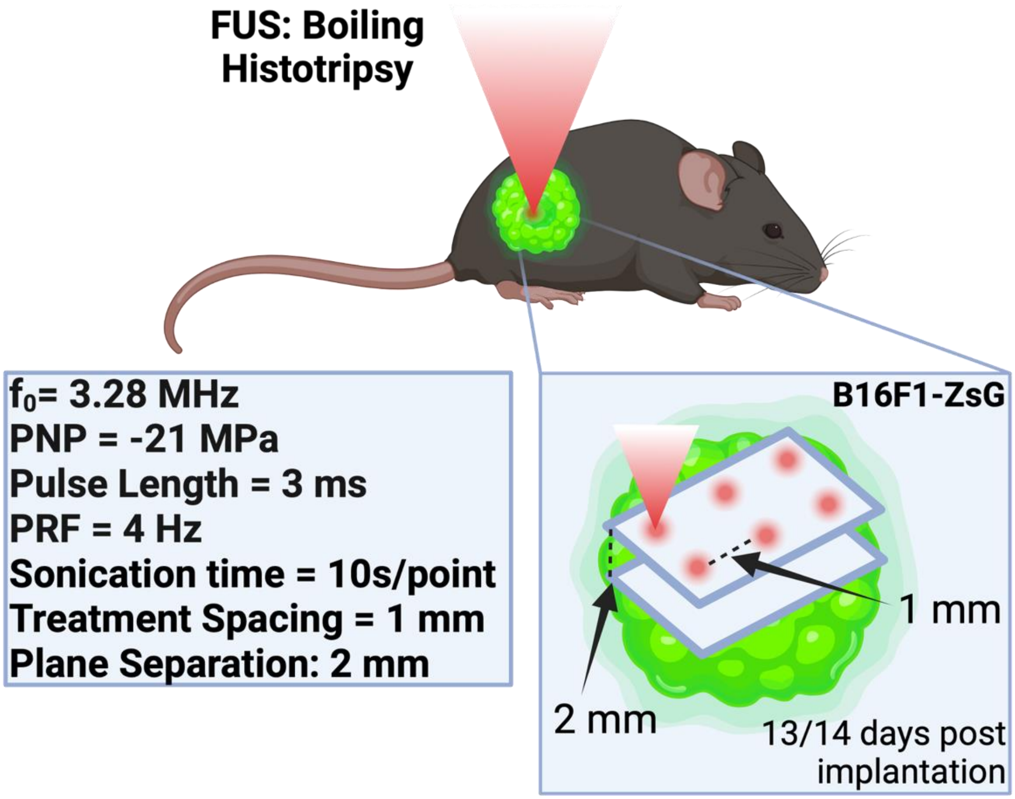

Figure S1. Schematic for boiling histotripsy treatment. Boiling histotripsy was applied with the following parameters: Operational frequency (f_0 = 3.28 MHz); peak-negative pressure (PNP = -21 MPa); pulse length (3 ms); pulse repetition frequency (PRF = 4 Hz); sonication time = 10 s/point; treatment spacing = 1 mm; plane separation = 2 mm. With this treatment scheme, ~20% of the tumor volume is within the -6dB focus during BH application.

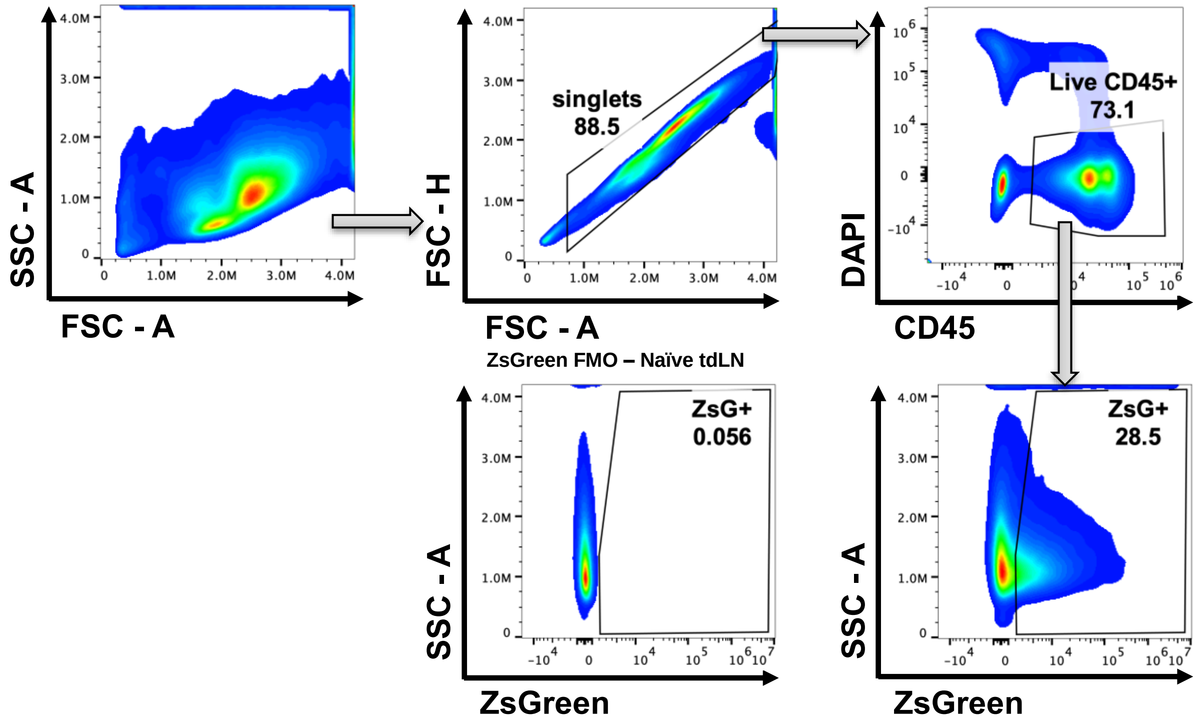

Figure S2. Gating strategy for flow cytometry analysis of Figure 1. ZsG-naïve mice were used as the gating control for ZsG positivity in LiveCD45^{+} immune cells.

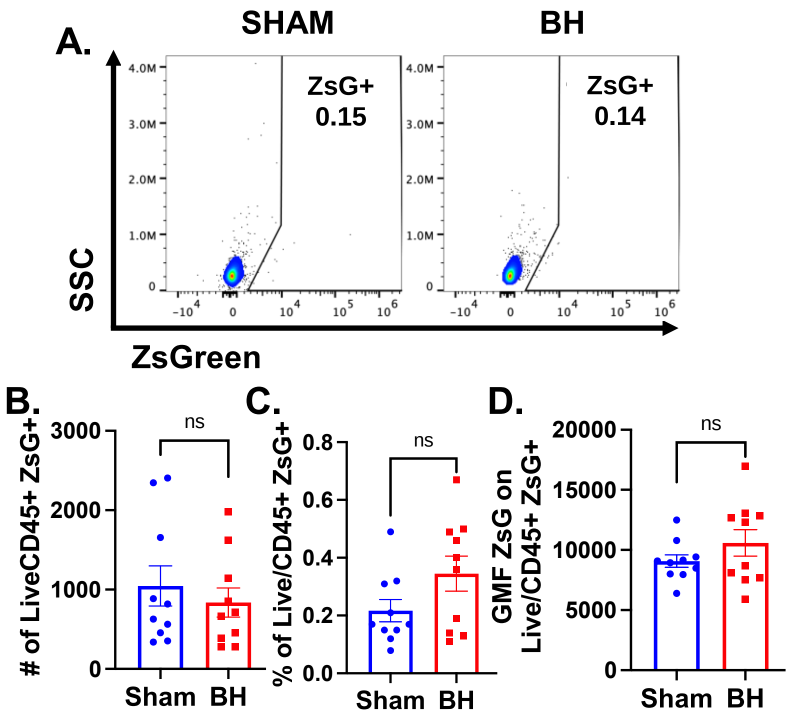

Figure S3. BH does not promote ZsG tumor antigen acquisition in non-tumor draining lymph nodes 24 hrs post treatment. A. Density scatter plots of side scatter vs ZsG indicating percent of Live/CD45^{+} cells that are ZsG^{+}. **B-D.** Summary of flow cytometry analysis. **B**) Number of LiveCD45^{+}ZsG^{+} cells. **C**) Percent of LiveCD45^{+} cells that are ZsG^{+}. **D**) Geometric mean fluorescent intensity (GMF) of ZsG on LiveCD45^{+}ZsG^{+} cells. n=10. Unpaired Welch's t-test: not significant. Means ± SEM.

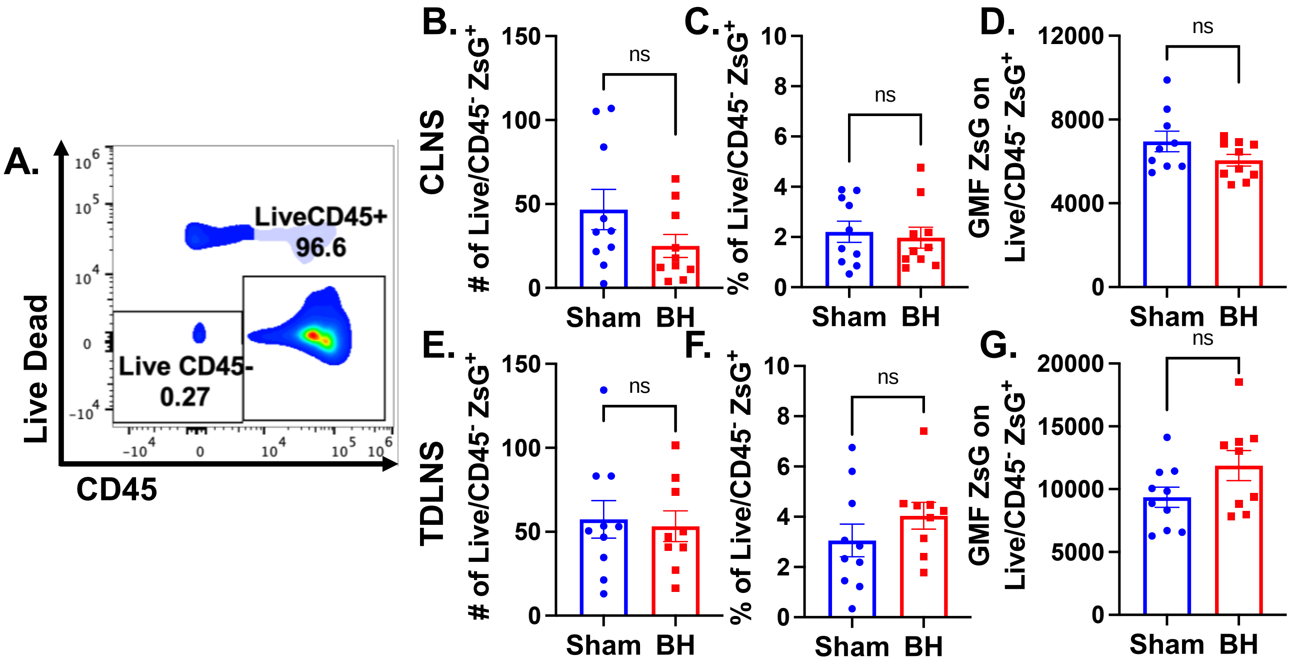

Figure S4. BH does not promote metastatic spread of melanoma 24 hrs after treatment. A. Density scatter plots of Live Dead vs CD45 indicating percent of singlet cells that are Live/CD45$^+$ and Live/CD45$^-$. The subsequent summary plots are in reference to ZsG$^+$ portion of LiveCD45$^-$ cells. **B, E)** Number of LiveCD45$^-$ZsG$^+$ cells in the contralateral and tumor draining lymph nodes. **C, F)** Percent of LiveCD45$^-$ cells that are ZsG$^+$ in CLNs and TDLN. **D, G)** The geometric mean fluorescent intensity (GMF) of ZsG on LiveCD45$^-$ZsG$^+$ cells in CLNs and TDLN**.** n=10. Unpaired Welch's t-test: not significant. Means ± SEM.

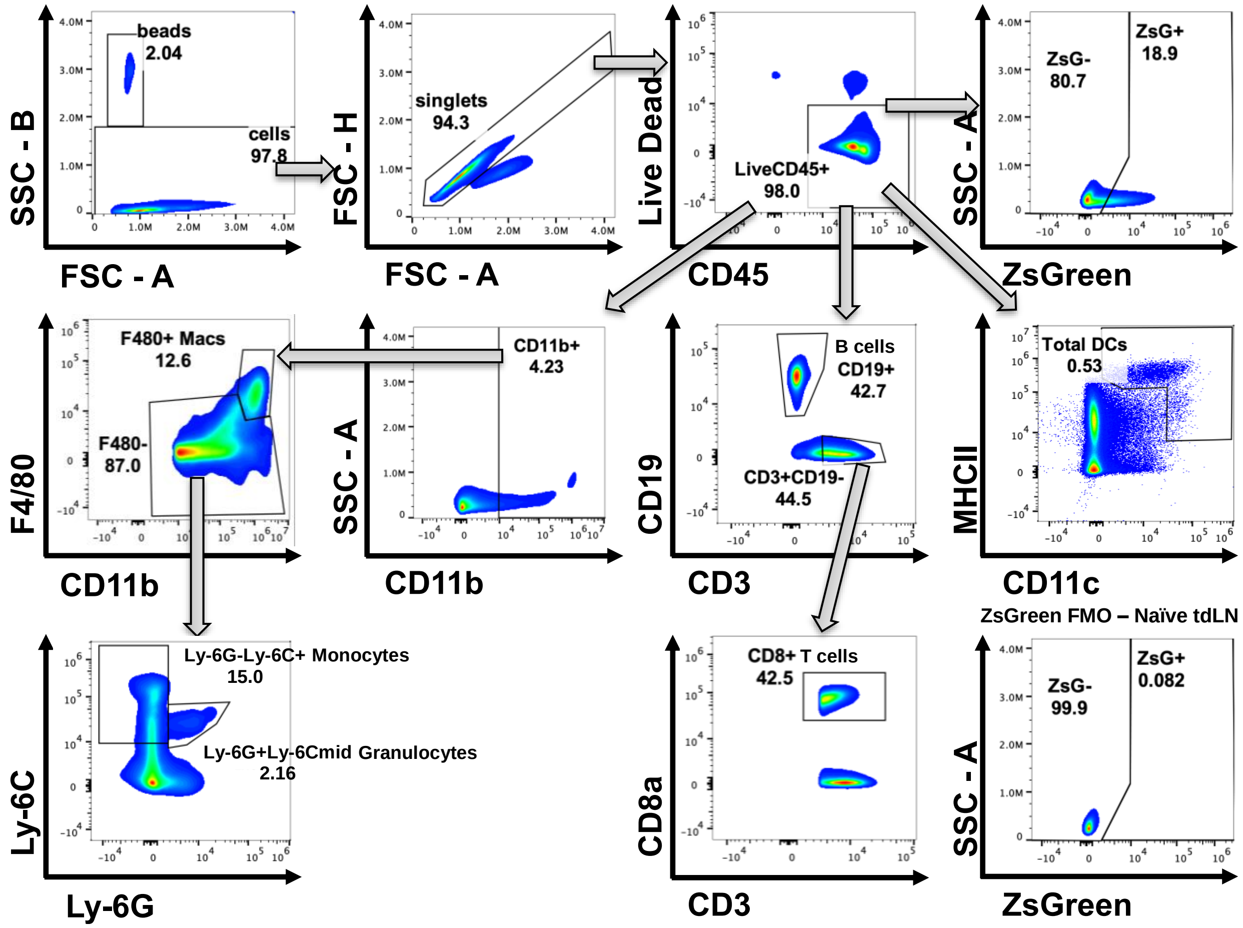

Figure S5. Gating strategy for flow cytometry analysis of Figure 2 and Figure S3. Gating strategy presented for the following immune cell subsets after cells and singlets: Macrophages ($LiveCD45^{+}CD11b^{+}F4/80^{+}$); Monocytes ($LiveCD45^{+}CD11b^{+}F4/80^{-}Ly\text{-}6G^{-}Ly\text{-}6C^{+}$); Granulocytes ($LiveCD45^{+}CD11b^{+}F4/80^{-}Ly\text{-}6G^{+}Ly\text{-}6C^{mid}$); B cells ($LiveCD45^{+}CD19^{+}$); CD8 T cells ($LiveCD45^{+}CD3^{+}CD19^{-}CD8^{+}$); Total DCs ($LiveCD45^{+}CD11c^{+}MHCII^{+}$). Gating strategy for $ZsGreen^{+}$ (ZsG^{+}) immune cells and the corresponding control from ZsG-naïve mice are outlined.

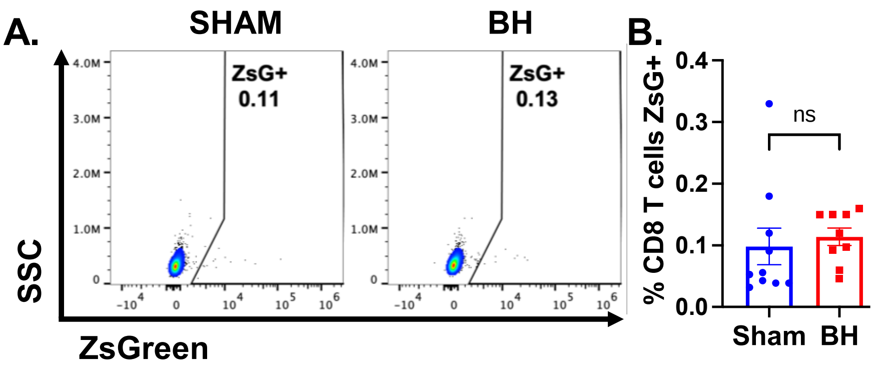

Figure S6. Negative control for ZsG fluorescence showing antigen acquisition by non-APC and non-phagocytic cells (CD8^{+} T cells). A. Density scatter plots of side scatter vs ZsG indicating percent of LiveCD45^{+}CD19^{-}CD3^{+}CD8^{+} T cells that are ZsG^{+}. **B.** Summary of flow cytometry analysis in **A**. n=10. Unpaired Welch's t-test: not significant. Means ± SEM.

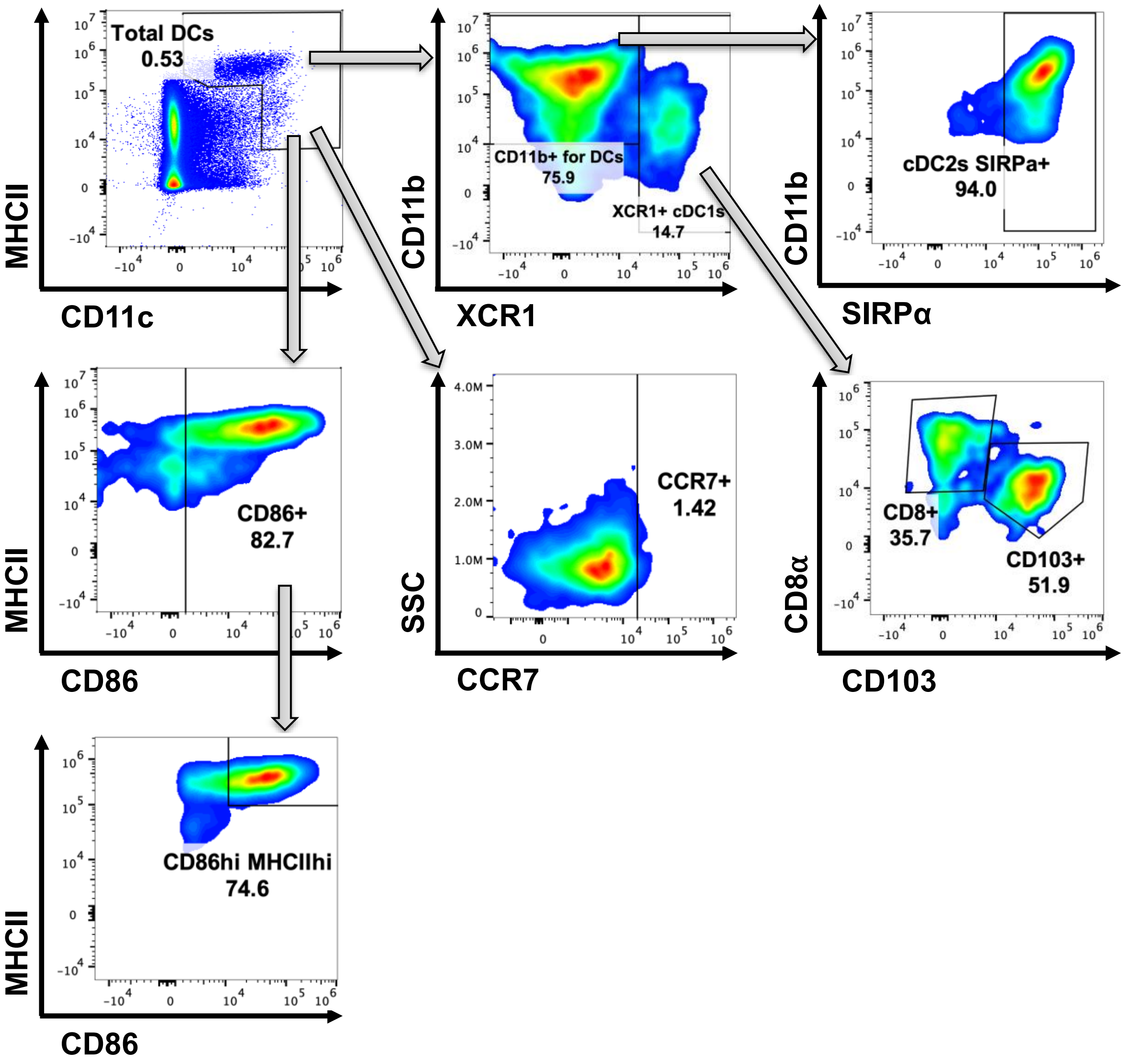

Figure S7. Gating strategy for flow cytometry analysis of cDCs and activated status. Gating strategy for the following DC (LiveCD45^{+}CD11c^{+}MHCII^{+}) subsets: cDC1s (XCR1^{+}), migratory cDC1s (XCR1^{+}CD103^{+}), tissue-resident cDC1s (XCR1^{+}CD8α^{+}CD103^{-}) and cDC2s (XCR1^{-}CD11b^{+}SIPRα^{+}). Activation of DC subsets was defined as a subset of CD86^{+}MHCII^{+}: CD86^{hi}MHCII^{hi}. CD86^{+} and CCR7^{+} gating strategies are shown.

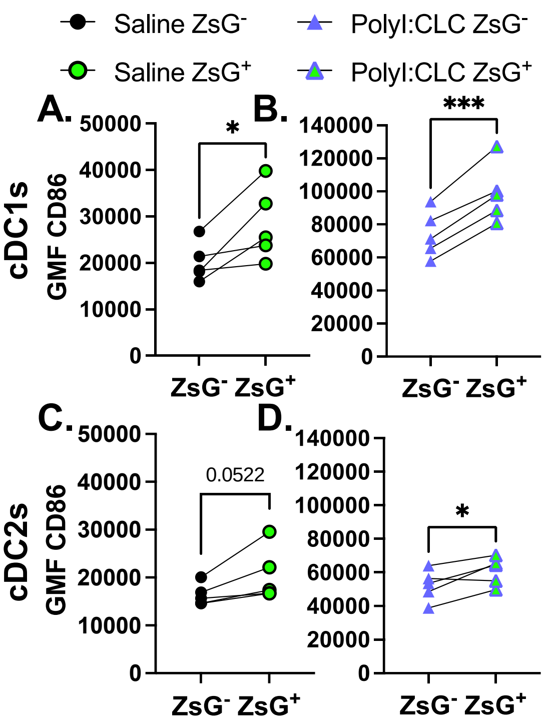

Figure S8. CD86 expression on cDCs is augmented by toll-like receptor 3 agonist polyI:CLC and ZsG. 400k B16F10-ZsG cells were inoculated in the right flanks of C57/Bl6 mice and tumors were treated with polyI:CLC (75 mg/0.1 mL saline I.P.) or saline (0.1 mL I.P.) 13 days post inoculation. Axial and brachial lymph nodes were harvested and pooled 24 h after saline or polyI:CLC injection. **A, B)** Geometric mean fluorescent (GMF) intensity of CD86 on cDC1s as a function of ZsG positivity. **C, D)** GMF intensity of CD86 on cDC2s as a function of ZsG. n = 10. Paired t-test: $*$ $p < 0.05$, $**$ $p < 0.01$, $***$ $p < 0.001$. Means ± SEM.

Table S1. Logistics model parameters.

Tumor Side	R^2		r		a	
	Sham (n = 11)	BH (n = 8)	Sham (n = 11)	BH (n = 8)	Sham (n = 11)	BH (n = 8)
**Ipsilateral**	0.9678 ± 0.0077 (0.9086 to 0.9933)	0.7993 ± 0.1038 (0.0902 to 0.9956)	$(4.66 \pm 2.58)x10^4$ (897 to $2.93x10^5$)	$(3.34 \pm 3.01)x10^6$ (0.0351 to $2.44x10^6$)	0.4626 ± 0.0263 (0.2888 to 0.5908)	0.4013 ± 0.0431 (0. 2465 to 0.5792)
**Contralateral**	0.9346 ± 0.0429 (0.5127 to 0.9975)	0.9393 ± 0.0298 (0.7489 to 0.9954)	$(1.43 \pm 0.51)x10^4$ (0.0094 to $4.77x10^4$)	$(3.31 \pm 1.25)x10^4$ (53.55 to 9.67 $x10^4$)	0.3625 ± 0.0370 (0.0907 to 0.5028)	0.3018 ± 0.0497 (0.0250 to 0.4304)
**Values are Means ± SEM. Parentheses show range, from minimum to maximum. Sample sizes indicated below group labels.2**						

Table S2. Key Resources Table.

REAGENTS	SOURCE	IDENTIFIER
Anti-mouse CD45 clone 30-F11 BUV395	BD	Cat# 564279; RRID: AB_2651134
Anti-mouse CD3 clone 145-2C11 BUV563	BD	Cat# 749277; RRID: AB_287352
Anti-mouse CD19 clone 1D3 BUV737	BD	Cat# 612781; RRID: AB_2870111
Anti-mouse F4/80 clone BM8 BV421	BioLegend	Cat# 123137; RRID: AB_2563102
Anti-mouse CD11c clone N418 eF450	Invitrogen	Cat# 48-0114-82; RRID: AB_1548654
Anti-mouse Ly6G clone 1A8 BV605	BioLegend	Cat# 127639; RRID: AB_2565880
Anti-mouse/rat XCR1 clone ZET BV650	BioLegend	Cat# 148220; RRID: AB_2566410
Anti-mouse CD103 clone 2E7 BV785	BioLegend	Cat# 121439; RRID: AB_2800588
Anti-mouse Ly-6C clone HK1.4 PE/Dazzle 594	BioLegend	Cat# 128044; RRID: AB_2566577
Anti-mouse CD172a (SIRPα^{+}) clone P84 PE	BioLegend	Cat# 144012; RRID: AB_2563550
Anti-mouse/human CD11b clone M1/70 PE/Cy5	BioLegend	Cat# 101209; RRID: AB_312792
Anti-mouse CD197 (CCR7) clone 4B12 PE/Cy7	Invitrogen	Cat# 25-1971-82; RRID: AB_469652
Anti-mouse CD86 (B7-2) clone GL1 APC	Invitrogen	Cat# 17-0862-82; RRID: AB_469419
Anti-mouse MHCII clone M5/114.15.2 AF700	Invitrogen	Cat# 56-5321-80; RRID: AB_494010
Anti-mouse CD8α clone 53-6.7 APC/Fire 750	BioLegend	Cat# 100766; RRID: AB_2572113
Fixable Live/Dead Blue	Invitrogen	Cat# L23105; UV Excitation
DAPI	Sigma-Aldrich	Cat# D9542
Fc Block	Invitrogen	Cat# 14-0161-86; RRID: AB_467135
Brilliant Stain Buffer	BD	Cat# 563794
FACS Lysis	BD	Cat# 349202
